# Kappa statistic to measure agreement beyond chance in free-response assessments

**DOI:** 10.1186/s12874-017-0340-6

**Published:** 2017-04-19

**Authors:** Marc Carpentier, Christophe Combescure, Laura Merlini, Thomas V. Perneger

**Affiliations:** 1 Division of Clinical Epidemiology, Geneva University Hospitals, and Faculty of Medicine, University of Geneva, Geneva, Switzerland; 2 Division of Radiology, Geneva University Hospitals, and Faculty of Medicine, University of Geneva, Geneva, Switzerland

**Keywords:** Reproducibility of results, Reliability (Epidemiology), Methodological Study, Biostatistics

## Abstract

**Background:**

The usual kappa statistic requires that all observations be enumerated. However, in free-response assessments, only positive (or abnormal) findings are notified, but negative (or normal) findings are not. This situation occurs frequently in imaging or other diagnostic studies. We propose here a kappa statistic that is suitable for free-response assessments.

**Method:**

We derived the equivalent of Cohen’s kappa statistic for two raters under the assumption that the number of possible findings for any given patient is very large, as well as a formula for sampling variance that is applicable to independent observations (for clustered observations, a bootstrap procedure is proposed). The proposed statistic was applied to a real-life dataset, and compared with the common practice of collapsing observations within a finite number of regions of interest.

**Results:**

The free-response kappa is computed from the total numbers of discordant (*b* and *c*) and concordant positive (*d*) observations made in all patients, as 2*d*/(*b* + *c* + 2*d*). In 84 full-body magnetic resonance imaging procedures in children that were evaluated by 2 independent raters, the free-response kappa statistic was 0.820. Aggregation of results within regions of interest resulted in overestimation of agreement beyond chance.

**Conclusions:**

The free-response kappa provides an estimate of agreement beyond chance in situations where only positive findings are reported by raters.

## Background

Good agreement between raters is a desirable property of any diagnostic method. Agreement is usually assessed by the kappa statistic [1], which quantifies by how much the observed agreement between raters exceeds agreement due to chance alone. The assessment of the kappa statistic requires the numbers of evaluations, both positive (or abnormal) and negative (or normal), to be known for all raters. This is not the case when raters report only positive findings and do not notify the number of negative findings. This situation can be referred to as the free-response paradigm [2]. It is a common situation in imaging procedures, where raters typically report positive findings, but do not list all negative observations for a given patient.

To date, the methods used to estimate the agreement corrected for chance of free-response assessments have all required a simplification of the data, so as to make negative findings explicit. One possibility is to analyze the data at the level of a patient, by rating a patient “positive” if at least one lesion is detected, but this causes an important loss of information. Another approach is to split the radiograph into regions of interest. Each region of interest is then assessed by all raters. Since negative ratings are explicitly notified, the number of regions of interest rated as negative by all raters is known and the standard kappa statistic can be computed. This approach reduces the loss of information compared with a single dichotomous rating per patient, but the regions of interest must be small and numerous enough to preserve clinical relevance. For instance, in a diagnostic study, Mohamed et al [3] defined 68 regions of interest per patient. Generally, constraining a free-response paradigm to a finite number of ratings (patient-level or region-level) causes a loss of information and may lead to overestimation of the agreement, because disagreements below the selected level of granularity are ignored.

The objective of the present paper is to propose a kappa statistic for free-response dichotomous ratings that does not require the definition of regions of interest or any other simplification of the observed data. This kappa statistic also takes into account within-patient clustering [4–6] of multiple observations made for the same patient.

## Methods

### Derivation of the free-response kappa

For two raters, the usual kappa statistic is (P_o_-P_e_)/(1-P_e_) where P_o_ is the proportion of observed concordant ratings and P_e_ is the expected proportion of concordant ratings due to chance alone. When the rating is dichotomous, data can be summarized in a 2 × 2 table. Let us denote by *a* the number of findings that are rated as negative by both raters, *b* and *c* the numbers of findings rated as positive by one rater but negative by the other, and *d* the number of findings rated as positive by both raters. There are therefore *a* + *d* concordant pairs of ratings and *b* + *c* discordant pairs among N pairs of observations. Assuming that observations are mutually independent, P_o_ is estimated by (a + d)/N and P_e_ by [(a + c) (a + b) + (c + d) (b + d)]/N^2^. Then, the kappa statistic (in this case, Cohen’s kappa) is given by:

1 $$ K=\frac{2\left( ad- bc\right)}{\left( b+ c\right) N+2\left( ad- bc\right)} $$

When patients can contribute more than one observation, data are clustered. Yang et al [7] proposed a kappa statistic obtained from the usual formula (P_o_-P_e_)/(1-P_e_) where P_o_ is a weighted average of the proportions of agreement over clusters (patients) and P_e_ is obtained from weighted averages of marginal proportions of ratings of each rater. With this approach, the kappa for clustered data has the same estimate as when clustering is ignored. Therefore the basic 2 × 2 table is also appropriate for the estimation of agreement for clustered data.

For free-response assessments, each rater reports only positive findings and the number *a* is unknown. It would be wrong to replace *a* by 0, as if the raters had not agreed on any negative observation; both the observed agreement and kappa would be underestimated. It would also be incorrect to simply replace *a* by the number of patients without any positive finding, because several potential lesion sites exist in each patient. Typically, *a* can be assumed to be high in imaging examinations, because each output displays a large number of anatomical or functional structures or substructures, each potentially positive or negative. Therefore, the number of positive findings in a given patient is usually small in comparison with the potential number of abnormalities that might occur.

We propose here a kappa statistic that describes Cohen’s kappa as *a* approaches infinity. The partial derivative of the kappa statistic defined in Eq. (1) with respect to *a* is:

$$ \frac{\partial \widehat{K}}{\partial a}=\frac{2\left( b+ c\right)\left( b+ d\right)\left( c+ d\right)}{{\left[\left( a+ b\right)\left( b+ d\right)+\left( a+ c\right)\left( c+ d\right)\right]}^2} $$

This partial derivative is positive, therefore the kappa statistic increases monotonously with *a*. Moreover this derivative has a null limit as *a* approaches infinity, which implies that the kappa statistic has a finite limit as *a* approaches infinity. We call this limit the free-response kappa (K_FR_). Per Eq. (1), K_FR_ is the ratio of two functions of *a*, *f* (*a*) = 2 (*ad*-*bc*) and *g* (*a*) = (*b* + *c*)(*a* + *b* + *c* + *d*) + 2 (*ad*-*bc*), both of which approach infinity as *a* approaches infinity, so that their ratio is indeterminate. By L’Hôpital rule, K_FR_ equals the limit of the ratio of the partial derivatives of *f* (*a*) and *g* (*a*) as *a* approaches infinity, which turns out to be

2 $$ {K}_{FR}=\frac{2 d}{b+ c+2 d} $$

### Properties of free-response kappa

K_FR_has several interesting properties. It does not depend on *a*, but only on the positive observations *b*, *c*, and *d*. Therefore the uncertainty about *a* does not preclude the estimation of agreement beyond chance if the number of negative findings can be considered very large.

When interpreting K_FR_, it is helpful to consider the numbers of ratings made by each rater individually. The first rater made *c* + *d* positive observations, and the second rater made *b* + *d* positive observations. Therefore the denominator *b* + *c* + *2d* is the total number of positive individual observations made by the 2 raters, *2d* is the number of positive observations made by either rater that were confirmed by the other, and *b* + *c* is the number of positive observations made by either rater that were not confirmed by the other. K_FR_ is thus the proportion of confirmed positive individual observations among all positive individual observations. A K_FR_ statistic of 0.5 means that half of the positive findings were confirmed by the other rater, which may be considered average, whereas 0.8 might be considered very good. This is in line with published interpretation guidelines for Cohen’s kappa [8].

When the data are clustered, K_FR_ can be obtained directly by collapsing the 2 × 2 tables of all clusters into a single 2 × 2 table and applying Eq. (2). The pooled K_FR_ is a weighted average of individual free-response kappa statistics of patients with at least one positive observation (each patient is indexed by *k*):

$$ {K}_{FR}={\displaystyle \sum_k}{v}_k\frac{2{d}_k}{b_k+{c}_k+2{d}_k} $$

where each weight ν_k_ represents the proportion of positive ratings in patient k among all positive ratings:

$$ {v}_k=\frac{b_k+{c}_k+2{d}_k}{b+ c+2 d} $$

It follows that patients without any detected lesions do not contribute to the estimate of K_FR_; their weight is zero. Therefore patient-level clustering does not need to be taken into account to compute K_FR_, and patients without positive finding can be ignored.

Of note, the equation for K_FR_ corresponds to the proportion of specific (positive) agreement as described by Fleiss [9]. While the equation is identical, the purpose and interpretation are different. For Fleiss, specific positive agreement (and also specific negative agreement) is a complementary statistic that enhances the interpretation of overall agreement. The omission of double negative observations is an a priori decision. Importantly, Fleiss is interested in observed agreement, not in agreement corrected for chance. Finally, Fleiss does not address the free-response context.

### Variance of the free-response kappa

Because K_FR_ is bound by 0 and 1, we first normalized the estimator by taking the logit of K_FR_, i.e. ln (K_FR_/(1- K_FR_)). The variance of the estimated logit (K_FR_), obtained by the delta method (Appendix 1) is:

3 $$ V a r\left( logit\left({K}_{FR}\right)\right)=\frac{\left( b+ c+ d\right)}{\left( b+ c\right) d} $$

Thus a confidence interval can be obtained for logit (K_FR_), and the lower and upper confidence bounds back-transformed to the original scale.

An alternative approach is to make use of the direct relationship between K_FR_ and the proportion of congruent pairs of observations among all available observations, p = d/(b + c + d). It is easily shown that K_FR_ = 2p/(1 + p). Therefore a 95% confidence interval can be obtained for p, using any available method for binomial proportions including exact methods, and the confidence bounds can be then back-transformed to the K_FR_ scale.

We have simulated the performance of three confidence interval methods for independent observations at K_FR_ values of 0.3, 0.5, 0.7, and 0.9, and for sample sizes (N = b + c + d) of 20, 50, 100, and 200. For each condition we generated 50’000 random samples from a binomial distribution with parameters N and p, where p was defined by K_FR_/(2-K_FR_), which is the inverse of the equation K_FR_ = 2p/(1 + p). For each sample we computed a 95% confidence interval using Eq. (3) for the logit of K_FR_, and also using 2 methods for the binomial parameter p that are appropriate for small samples in which asymptotic estimation methods may yield incorrect results: the Agresti-Coull method [10], and the Clopper-Pearson method [11]. For each situation we report the mean simulated value of K_FR_, the proportion of confidence intervals that include the true value, and the mean width of the confidence intervals.

All three methods performed well (Table 1). Confidence intervals based on Eq. (3) had a lowered coverage (0.932) when the sample size and K_FR_ were both small. This is because in this case 2% of the samples were degenerate (d = 0 or d = N), and Eq. (3) could not be applied (if we had excluded these samples the coverage would have been 0.951). The Clopper-Pearson method produced the highest levels of coverage, but this was at the expense of unnecessarily wide confidence intervals. Confidence intervals were narrower for Eq. (3) and for the Agresti-Coull method.

**Table 1:** Simulations of the coverage and mean width of 95% confidence intervals for the free-response kappa at selected sample sizes (20, 50, 100, 200) and values of kappa (0.3, 0.5, 0.7, 0.9), using three methods: delta method (Eq. 3), Agresti-Coull confidence limits, and Clopper-Pearson confidence limits

Simulation parameters		Mean observed K_FR_	Degenerate sample^a^(d = 0 or d = N)	Coverage of 95% confidence interval			Mean width of 95% confidence interval		
N	K_FR_			Logit delta method (Equation 3)	Agresti-Coull method	Clopper-Pearson method	Logit delta method (equation 3)	Agresti-Coull method	Clopper-Pearson method
20	0.3	0.291	0.020	0.932	0.952	0.966	0.446	0.444	0.473
	0.5	0.491	<0.001	0.944	0.944	0.969	0.426	0.419	0.471
	0.7	0.693	0	0.957	0.957	0.976	0.354	0.345	0.392
	0.9	0.897	0.019	0.964	0.981	0.964	0.224	0.218	0.235
50	0.3	0.297	<0.001	0.962	0.962	0.962	0.293	0.294	0.314
	0.5	0.497	0	0.949	0.949	0.965	0.284	0.281	0.305
	0.7	0.697	0	0.953	0.936	0.968	0.230	0.227	0.246
	0.9	0.899	<0.001	0.958	0.958	0.974	0.134	0.134	0.142
100	0.3	0.298	0	0.954	0.954	0.954	0.211	0.212	0.223
	0.5	0.498	0	0.945	0.945	0.968	0.204	0.203	0.215
	0.7	0.698	0	0.946	0.946	0.966	0.164	0.163	0.172
	0.9	0.899	0	0.948	0.948	0.963	0.093	0.093	0.098
200	0.3	0.299	0	0.947	0.947	0.959	0.151	0.151	0.157
	0.5	0.499	0	0.948	0.948	0.957	0.146	0.145	0.151
	0.7	0.699	0	0.952	0.952	0.952	0.116	0.116	0.120
	0.9	0.900	0	0.957	0.957	0.957	0.065	0.065	0.068

Each simulation based on 50′000 replicates

^a^Logit delta method not applicable. These simulations were treated as cases of non-coverage, and were not used for computation of the width of the confidence interval for this method

Of note, the mean values of observed K_FR_ were slightly below the parameter values, especially at low sample sizes. This is because we simulated with a fixed parameter p, and K_FR_ = 2p/(1 + p) is a concave function. By Jensen’s inequality, the expectation of a concave function of p (i.e., the mean observed K_FR_) will be then less than the function of the expectation of p (i.e., the K_FR_ that corresponds to the parameter p).

To be valid, these estimation methods require observations to be mutually independent. This may apply in some circumstances: e.g., if a paired screening test is applied to a large population, and only those with at least one positive result are referred for further investigation. But for most imaging procedures data are naturally clustered within patients. Then the proposed asymptotic variance of K_FR_ would be biased. In presence of clustering, a bootstrap procedure can be used to obtain a confidence interval (see Appendix 2).

## Results: case study

A recent study [12] examined the inter-rater agreement for a specific Magnetic Resonance Imaging (MRI) sequence among 84 children who underwent a full body MRI for any reason at a large public hospital. Two radiologists, blinded to each other’s assessments, reported all lesions they identified in each patient. A third radiologist linked these independent readings and identified all unique lesions, and therefore concordant and discordant diagnoses. In total 249 distinct lesions were identified in 58 children (the other 26 had a normal MRI); 76 were discordant and 173 concordant (Table 2).

**Table 2:** Contingency table of matched ratings in the Magnetic Resonance Imaging study

		Second Rater		
		Negative	Positive	Total
First Rater	Negative	unspecified	19	
	Positive	57	173	230
	Total		192	249

**Fig. 1:**
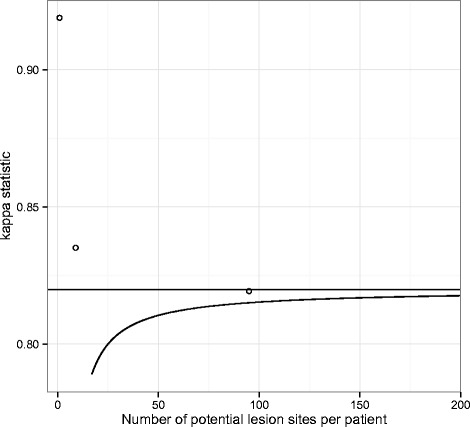
Estimates of the kappa statistic in full-body Magnetic Resonance Imaging examinations of 84 children. The curve represents the kappa statistic when the number of potential lesion sites per patient ranges from 17 to 200. The horizontal line represents the free-response kappa (0.820). Circles show the kappa statistic computed for regions of interest at 3 levels of grouping (0.919 at the patient level, 0.835 for 9 regions of interest per patient, and 0.819 for 95 regions of interest per patient)

If we assumed that no double negative ratings existed the kappa statistic would be−0.129 (95% confidence interval (95% CI),−0.208,−0.058; all confidence intervals were obtained using the bootstrap procedure described in Appendix 2); the observed agreement would be lower than what would be expected from chance. What would be a reasonable estimate for *a*? The highest number of detected lesions was 17 for one patient, which indicates that the potential number of lesion sites per patient was at least 17. Therefore, a patient with no lesions should count for at least 17 double negative ratings. In this case the total number of sites evaluated would be 84×17 = 1428 and, by subtraction, *a* would be 1179 and the kappa statistic 0.789 (95% CI: 0.696–0.868), well above−0.129. However, 95 distinct lesion sites were identified in the sample. If the potential number of lesion sites per patient was 95, the total number of sites would be 7980, *a* would be 7731, and the kappa statistic 0.815 (0.731, 0.884). But the universe of possible lesions can be assumed to be larger than the few observed in this sample. Figure 1 (solid line) shows the kappa statistics when *a* ranges from 17 to 200 per patient; the horizontal line corresponds to the free-response kappa of 0.820 (0.737, 0.888). This example shows that kappa is underestimated when potentially unlimited negative ratings are ignored or undercounted.

The effect of aggregating the ratings over regions of interest goes in the opposite direction (Fig. 1, circles). A region of interest can be defined by a patient (in which case the presence of any lesion renders the patient “positive”), but more often by an arbitrary division of space, or by a specific anatomical structure. We consider here 3 levels of granularity: the patient level (1 region of interest), an intermediate level where lesions are grouped according to an anatomical typology (9 regions of interest; for example: long bones, joints, soft tissues…) and the original level of the ratings (95 regions of interest; for example: left femoral bone, joint effusion of the right knee, retroperitoneal mass…). Table 3 shows the corresponding 2 × 2 tables.

**Table 3:** Contingency tables of matched ratings for three different levels of regions of interest

Patient level		Second Rater		
		Negative	Positive	Total
First Rater	Negative	26	1	27
	Positive	2	55	57
	Total	28	56	84
Intermediate level (9 regions per ratient)		Second Rater		
		Negative	Positive	Total
First Rater	Negative	640	8	648
	Positive	21	87	108
	Total	661	95	84×9
Detailed level (95 regions per ratient)		Second Rater		
		Negative	Positive	Total
First Rater	Negative	7743	18	7761
	Positive	53	166	219
	Total	7796	184	84×95

At the patient level, of 84 mutually independent ratings, 81 are concordant (55 patients for whom each rater found at least one abnormality, and 26 double negatives), and 3 discordant (patients for whom one rater found a lesion and the other found none). In this case the kappa statistic is 0.919 (0.816, 1.00). At the intermediate level (9 regions of interest per patient), there are 29 discordant ratings, 727 concordant ratings, and the kappa statistic equals 0.835 (0.763, 0.901). Finally at the detailed level (95 regions of interest per patient), there are 71 discordant findings, 7909 concordant findings, and the kappa statistic is 0.819 (0.738, 0.889). This result is virtually indistinguishable from the free-response kappa of 0.820, which assumed an infinite number of potential findings.

## Discussion

Situations in which only positive findings are explicit are frequent in imaging procedures. Images can cover large areas or even the whole body, and identify multiple abnormalities, such as metastases, plaques in multiple sclerosis, or stenoses along the coronary system. In many cases the universe of possible findings (abnormalities or lesions) is very large and cannot be enumerated. The lack of a specific number of double-negative observations precludes the use of the classic formulation of the kappa statistic.

In this paper, we propose a variant of the kappa statistic that relies on the properties of the classic kappa statistic when the number of negative ratings can be considered large. In that case, agreement does not depend on the unknown data and can be estimated from positive findings only. This free-response kappa corresponds to the proportion of all confirmed individual positive ratings (*2d*) among all positive individual ratings (*b* + *c* + *2d*).

Unlike simplifications that circumvent the free-response paradigm, the free-response kappa statistic only uses the available data at the level at which ratings – and specifically, the decisions about what constitutes agreement or disagreement – were made. It requires neither an enumeration of all possible lesion sites, nor a reduction of the data by defining regions of interest. On the contrary, for the free-response kappa, the more precise the ratings are, the more they conform to the assumption a non-finite universe of lesions.

The validity of the free-response kappa relies upon an accurate definition of concordant and discordant findings. This is true for any agreement study, but for Cohen’s kappa, e.g. when regions of interest are defined, pairing is straightforward because it follows the definition of the regions or objects of study. The free-response paradigm requires that observations from 2 raters be classified as concordant or discordant. This must be planned carefully when designing the study and defining the rating procedures. Typically, such a study is done in two steps: first, 2 independent raters assess the images, and then a third independent rater identifies concordant pairs. Therefore the concordance of the 2 descriptions is determined by a human observer, who may be prone to error. For this reason the descriptive system used by the raters should be as detailed as required for clinical management, and fully standardized to facilitate the decisions regarding agreement.

### Assumption of infinity

The notion of an infinite number of potential lesions may appear excessive or unrealistic. However, when one considers the number of anatomical structures in the human body, multiplied by the number of study participants, this is not far fetched. Furthermore, once the possible number of double negative observations in the study (i.e., in all participants) exceeds a few thousand, K_FR_ has reached its asymptote and does not change meaningfully if this number is further increased. Nevertheless K_FR_ can be considered as an upper bound on agreement corrected for chance.

The requirement of a large number of potential lesions is not fulfilled in all imaging studies. If one is interested in measuring agreement on the chest X-ray performed to rule out iatrogenic pneumothorax after a central venous catheter insertion, there is one diagnosis and only a few radiologic signs to consider. In this case, the number of clinically relevant normal findings is limited and the free-response kappa would not be appropriate. Then, and more generally when it is reasonable to specify the number X of potential abnormalities that can be identified, it is reasonable to use X to infer the number of double negatives, as a = X-b-c-d, and to obtain the standard kappa statistic.

### Clustering of observations

For most imaging procedures, each patient can contribute several positive findings, and data are naturally clustered within patients. Clustering does not influence the computation of the free-response kappa, but must be taken into account for the computation of the standard error. Importantly, the global free-response kappa is a weighted average of within-cluster kappa statistics, with weights proportional to *b*_*k*_ + *c*_*k*_ + *2d*_*k*_, the total number of positive ratings in a cluster (ignoring pairing). This decomposition holds for any partition of the data and could be done for any covariate, e.g., to compare agreement beyond chance in obese versus non-obese patients, or for skeletal lesions versus lesions in soft tissues.

When observations are independent, confidence intervals can be computed using several methods, compared in Table 1. For clustered data, a common situation in radiology, we propose a bootstrap-based approach. We sampled patients (with replacement), and used all observations from any selected patient [13, 14]. We reasoned that this represented best the role of sampling variability in imaging studies: a patient is a “random” factor, but a lesion within a patient is not. Nevertheless, alternative methods for the estimation of K_FR_ should be explored in future studies. Future developments should also address the generalization of free-response kappa to multiple raters, and to ordinal ratings.

## Conclusions

We have proposed a kappa statistic that is appropriate for free-response assessments, and discussed its properties. This statistic may be particularly useful for imaging studies.

## Appendix 1

### Variance of the free-response kappa K_FR_ for independent data

The logit of the free-response kappa statistic is function of the number of discordant pairs of ratings, denoted by *x* = *b* + *c*, and of the number of concordant pairs of ratings, *d*:

$$ logit\left({\widehat{K}}_{FR}\right)= \ln \left(\frac{2 d}{x}\right)= g\left( x, d\right) $$

The variance of a function of random variables can be approximated using a first-order Taylor expansion [15]:

$$ V a r\left( logit\left({\widehat{K}}_{FR}\right)\right)\approx {\left(\frac{\partial g}{\partial x}\right)}^2 V a r(X)+{\left(\frac{\partial g}{\partial d}\right)}^2 V a r(D)+2\frac{\partial g}{\partial x}\frac{\partial g}{\partial y} C o v\left( X, D\right) $$

where *X* is a binomial variable Bin (p_*X*_, *x* + *d*) and *D* is a binomial variable Bin (1-p_*X*_, *x* + *d*) such that *X* + *D* is the total number of pairs of ratings (*b* + *c* + *d*). The derivatives and the estimated variances can be calculated from observations:

$$ \frac{\partial g}{\partial x}=-\frac{1}{x} $$

$$ \frac{\partial g}{\partial d}=\frac{1}{d} $$

$$ V a r(X)=\frac{ x d}{x+ d} $$

$$ V a r(D)=\frac{ x d}{x+ d} $$

$$ C o v\left( X, D\right)=-\frac{ x d}{x+ d} $$

Finally, the variance of the logit of the free-response kappa can be approximated by:

$$ V a r\left( logit\left({\widehat{K}}_{FR}\right)\right)\approx \frac{\left( x+ d\right)}{xd} $$

Lower and upper bounds of the 95% confidence interval of the transformation are:

$$ L B= logit\left({\widehat{K}}_{FR}\right)-1.96\sqrt{\frac{\left( x+ d\right)}{xd}};\  U B= logit\left({\widehat{K}}_{FR}\right)+1.96\sqrt{\frac{\left( x+ d\right)}{xd}} $$

And the 95% confidence interval on the original scale is obtained with the inverse transformation of the logit function:

$$ \frac{{\mathrm{e}}^{\mathrm{LB}}}{1+{\mathrm{e}}^{\mathrm{LB}}};\ \frac{{\mathrm{e}}^{\mathrm{UB}}}{1+{\mathrm{e}}^{\mathrm{UB}}} $$

## Appendix 2

### Standard error of the free-response kappa K_FR_ and 95% confidence interval for clustered data

A bootstrap procedure has been proposed by Kang et al. to estimate the standard error of the kappa statistic when data are clustered [13]. This procedure is applicable to the free-response kappa statistic:

1. Randomly sample *R* clusters (patients) with replacement from the original data set; *R* should be equal to the number of available patients, including patients with no abnormal finding
2. Calculate the kappa statistic ${\widehat{K}}_{FR}^b$ collapsing data from the selected clusters (patients) and using Equation (1)
3. Repeat steps 1 and 2 *B* times to generate *B* bootstrap samples and *B* free-response kappa statistics.

From the distribution of ${\widehat{K}}_{FR}^b$, there are several ways to obtain a 95% confidence interval. A standard normal confidence interval could be computed using the standard error of ${\widehat{K}}_{FR}^b$, or preferably the standard error of the logit of ${\widehat{K}}_{FR}^b$ for the reasons discussed previously. The percentile-based interval and the bias-corrected accelerated interval are alternative approaches [16]. These methodological considerations are beyond the scope of this paper, and here we only report the percentile-based intervals (percentiles 2.5 and 97.5).

## Abbreviations

CI: Confidence interval
K_FR_: Free-response kappa
Logit (x): Ln (x/(1-x))
MRI: Magnetic Resonance Imaging

## References

[1] Kundel HL, Polansky M (2003). Measurement of observer agreement. Radiology.

[2] Chakraborty DP. A brief history of free-response receiver operating characteristic paradigm data analysis. Acad Radiol. 2013;20:915–9. 10.1016/j.acra.2013.03.001 PMC3679336 23583665

[3] Mohamed ASR, Ruangskul MN, Awan MJ, et al. Quality assurance assessment of diagnostic and radiation therapy–simulation CT image registration for head and neck radiation therapy: anatomic region of interest–based comparison of rigid and deformable algorithms. Radiology. 2014;274:752–63. 10.1148/radiol.14132871 PMC4358813 25380454

[4] Gönen M, Panageas KS, Larson SM. Statistical issues in analysis of diagnostic imaging experiments with multiple observations per patient. Radiology. 2001;221:763–7. 10.1148/radiol.2212010280 11719674

[5] Genders TSS, Spronk S, Stijnen T, Steyerberg EW, Lesaffre E, Hunink MGM. Methods for calculating sensitivity and specificity of clustered data: a tutorial. Radiology. 2012;265:910–6. 10.1148/radiol.12120509 23093680

[6] Levine D, Bankier AA, Halpern EF. Submissions to *Radiology*: our Top 10 list of statistical errors. Radiology. 2009;253:288–90.

[7] Yang Z, Zhou M (2014). Kappa statistic for clustered matched-pair data. Stat Med.

[8] Landis JR, Koch GG (1977). The measurement of observer agreement for categorical data. Biometrics.

[9] Fleiss JL. Statistical Methods for Rates and Proportions. Second ed. New York: Wiley, John and Sons, Incorporated; 1981. p. 214–5.

[10] Agresti A, Coull BA (1998). Approximate is better than “exact” for interval estimation of binomial proportions. Am Stat.

[11] Clopper CJ, Pearson ES (1934). The use of confidence or fiduacial limits illustrated in the ase of the binomial. Biometrika.

[12] Merlini L, Carpentier M, Ferrey S, Anooshiravani M, Poletti PA, Hanquinet S. Whole-body MRI in children: Would a 3D STIR sequence alone be sufficient for investigating common paediatric conditions? A comparative study. Eur Radiol. 2017;88:155–62. 10.1016/j.ejrad.2017.01.014 28189202

[13] Kang C, Qaqish B, Monaco J, Sheridan SL, Cai J (2013). Kappa statistic for clustered dichotomous responses from physicians and patients. Stat Med.

[14] Field CA, Welsh AH (2007). Bootstrapping clustered data. J R Stat Soc Ser B Stat Methodol.

[15] Casella G, Berger RL (1990). Statistical Inference.

[16] Efron B, Tibshirani RJ (1994). An Introduction to the Bootstrap.

